# Improved Performance of Electron Blocking Layer Free AlGaN Deep Ultraviolet Light-Emitting Diodes Using Graded Staircase Barriers

**DOI:** 10.3390/mi12030334

**Published:** 2021-03-21

**Authors:** Barsha Jain, Ravi Teja Velpula, Moulik Patel, Sharif Md. Sadaf, Hieu Pham Trung Nguyen

**Affiliations:** 1 Department of Electrical and Computer Engineering, New Jersey Institute of Technology, Newark, NJ 07102, USA; bj226@njit.edu (B.J.); rv366@njit.edu (R.T.V.); map244@njit.edu (M.P.); 2 Centre Energie, Matériaux et TéléCommunications, Institut National de la Recherche Scientifique (INRS), 1650 Boulevard Lionel-Boulet, Varennes, QC J3X 1S2, Canada; sharif.sadaf@inrs.ca

**Keywords:** AlGaN light-emitting diodes, electron-blocking layer, positive sheet polarization charges, thermal velocity, graded staircase quantum barriers (GSQBs)

## Abstract

To prevent electron leakage in deep ultraviolet (UV) AlGaN light-emitting diodes (LEDs), Al-rich *p*-type Al_x_Ga_(1−x)_N electron blocking layer (EBL) has been utilized. However, the conventional EBL can mitigate the electron overflow only up to some extent and adversely, holes are depleted in the EBL due to the formation of positive sheet polarization charges at the heterointerface of the last quantum barrier (QB)/EBL. Subsequently, the hole injection efficiency of the LED is severely limited. In this regard, we propose an EBL-free AlGaN deep UV LED structure using graded staircase quantum barriers (GSQBs) instead of conventional QBs without affecting the hole injection efficiency. The reported structure exhibits significantly reduced thermal velocity and mean free path of electrons in the active region, thus greatly confines the electrons over there and tremendously decreases the electron leakage into the *p*-region. Moreover, such specially designed QBs reduce the quantum-confined Stark effect in the active region, thereby improves the electron and hole wavefunctions overlap. As a result, both the internal quantum efficiency and output power of the GSQB structure are ~2.13 times higher than the conventional structure at 60 mA. Importantly, our proposed structure exhibits only ~20.68% efficiency droop during 0–60 mA injection current, which is significantly lower compared to the regular structure.

## 1. Introduction

The AlGaN-based ultraviolet (UV) light-emitting diodes (LEDs) offer tremendous potential for a wide range of applications, including air/water purification, surface disinfection, biochemical sensing, cancer cell elimination, and many more [1]. These UV LEDs have the potential to replace the bulky and toxic conventional UV lamps due to advantages like environment-friendly material composition, longer life-time, low power consumption due to low DC drive voltage, compact in size, and tunable emission across the UV region from ~200 nm to ~365 nm [2]. Nevertheless, the external quantum efficiency (EQE) and light output power of AlGaN deep UV-LEDs are still low due to several challenges. For instance, strong induced polarization fields and quantum-confined Stark effect (QCSE) contribute significantly to the separation of electron and hole wave functions, leading to reduced carrier confinement and radiative recombination in the device active region. Subsequently, the electron overflow, which acts as one of the primary reasons for efficiency droop is increased [3].

To eliminate the electron overflow, a *p*-doped Al-rich electron blocking layer (EBL) has been introduced between the active region and *p*-region [4]. This could mitigate the electron leakage to only an extent. However, hole injection efficiency is affected owing to the formation of positive polarization sheet charges at the interface of the last quantum barrier and EBL [5]. Moreover, as the EBL is Al-rich, Mg doping efficiency gets affected because of high acceptor activation energy, compensation by nitrogen vacancies, increased hole scattering, and limited acceptor solubility [6]. To address the above-mentioned problems, QW or EBL is re-engineered using different approaches [7,8,9,10,11]. This could partially reduce the challenges generated by the integration of the EBL, but it is always desired to improve the LED efficiency by eliminating the EBL layer. In this regard, different EBL-free LED designs have been studied for III-nitride semiconductor LEDs. Linear graded quantum barrier (QB)-based EBL-free AlGaN UV LEDs with similar optical performance compared to conventional EBL LEDs [12], strip-in-a-barrier AlGaN UV LEDs without EBL with remarkably high performance compared to regular EBL LEDs [13], band engineered EBL-free AlInN UV LEDs [14], lattice-matched InGaN/AlInN/InGaN QB visible LEDs without EBL [15], EBL-free coupled quantum wells (QWs) based InGaN/GaN nanowire LED for white light emission [16] are some of the reported studies. However, to-date, a study on high-performance EBL free AlGaN deep UV LEDs is limited. Therefore, it is necessary to further engineer the device structures to achieve high-performance without using EBL that obviates the EBL-related problem.

In this work, we have designed EBL-free AlGaN UV-LEDs with the utilization of the graded staircase quantum barriers (GSQBs) in the active region. There are available studies on staircase barriers for the visible region LEDs with and without the usage of EBL [17,18,19]. However, the reported UV-LED study using staircase QB structure contains the EBL, due to which the above-mentioned challenges related to EBL remain [20]. In our proposed structure, due to the incorporation of GSQBs, kinetic energy and the velocity of the electrons entering the active region reduces, thereby reducing the electron mean free path and improving the electron confinement in the active region. Besides, the effective conduction band barrier height (CBBH) of each QB in the active region gradually increases along the growth direction in the proposed structure that effectively blocks the electron overflow into the *p*-region without using EBL. As a result, the non-radiative recombination in the *p*-region could be dramatically reduced. Since the proposed structure does not require an EBL, it eliminates the formation of positive polarization sheet charges at the heterointerface of the last QB and EBL. Moreover, such specially designed QBs reduce the QCSE in the active region, thereby improves the electron and hole wavefunction overlap. As a result of the above-mentioned advantages, internal quantum efficiency (IQE) and output power of the proposed structure are notably improved with lower efficiency droop as compared to the conventional structure.

## 2. Device Structure and Parameters

**Figure 1:**
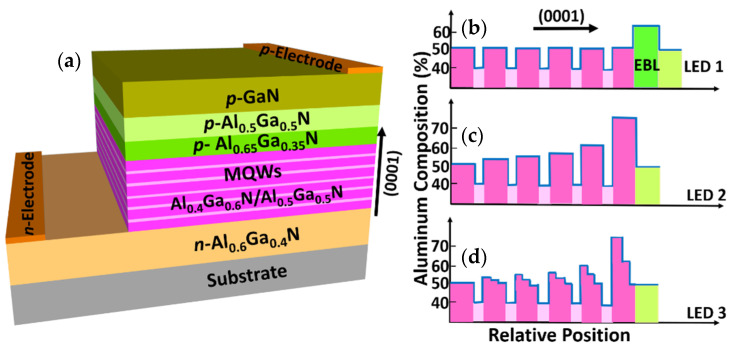
(**a**) Schematic diagram of light-emitting diode (LED) 1, Al composition (%) profile related to the conduction band of (**b**) LED 1 with conventional quantum barriers (QBs), (**c**) LED 2 with uniformly increasing Al composition in QBs, and (**d**) LED 3 with the proposed graded staircase quantum barrier (GSQB) structure.

Firstly, to validate our device model and parameters, we have considered the conventional EBL-based AlGaN deep UV LED structure grown on a *c*-plane AlN template with ~284 nm wavelength emission as a reference structure and denoted with LED 1. This study was experimentally reported by Yan et al. [21]. LED 1 consists of a 3 μm *n-*Al_0.6_Ga_0.4_N layer (Si doping concentration: 5 × 10^18^ cm^−3^), succeeded by an active region, followed by a 20 nm *p-*Al_0.65_Ga_0.35_N EBL (Mg doping concentration: 2 × 10^19^ cm^−3^), then capped by a 50 nm *p-*Al_0.5_Ga_0.5_N hole injection layer (Mg doping concentration: 2 × 10^19^ cm^−3^), and finally a 120 nm *p-*GaN contact layer (Mg doping concentration: 1 × 10^20^ cm^−3^). The active region comprises of five intrinsic 3 nm Al_0.4_Ga_0.6_N QWs sandwiched between six intrinsic 12 nm Al_0.5_Ga_0.5_N QBs. The schematic diagram of LED 1 is presented in Figure 1a and the Al composition (%) profile related to the conduction band energy diagram of LED 1 is shown in Figure 1b. The mesa area of the deep UV LED chip is 400 µm × 400 µm. As illustrated in Figure 1c, LED 2 has the same structure as LED 1 except the QBs, where the Al composition of the QBs is gradually increasing from QB_2_ to QB_6_ as 0.51, 0.54, 0.57, 0.60, and 0.75, respectively. The proposed structure referred to as LED 3 is identical to LED 2 with the replacement of GSQBs instead of uniform Al composition QBs. As depicted in Figure 1d, 12 nm thick each QB consists of Al_x_Ga_(1−x)_N (4 nm)/Al_(x + 0.5)/2_Ga_1−(x + 0.5)/2_N (4 nm)/Al_0.5_Ga_0.5_N (4 nm) step layers. The Al composition (x) in the last five QBs is 0.51, 0.54, 0.57, 0.60, and 0.75, respectively. The x values are chosen by carefully optimizing the structure, similar to our previous study [13].

In this study, the above-mentioned LED structures are numerically studied using the Advanced Physical Models of Semiconductor Devices (APSYS) tool. The energy bandgap of GaN and AlN are estimated using the Varshni formula [22]

(1) $$E_{g}(T)=E_{g}(0)-\frac{aT^{2}}{b+T}$$

where *E_g_*(*T*) and *E_g_*(0) are the energy bandgap at temperatures *T* and 0 K, respectively. *a* and *b* are material constants. The values of *a, b*, and *E_g_*(0) for GaN are 0.909 meV/K, 830 K, and 3.507 eV [23]. The corresponding values for AlN are 1.799 meV/K, 1462 K, and 6.23 eV, respectively [23]. The band offset ratio and bowing parameter for AlGaN are taken as 0.67/0.33 and 0.94 eV, respectively [24]. The carrier mobility is estimated using the Cauchy-Thomas approximation [25] and the energy band diagrams of LED structures are calculated by using 6 × 6 *k.p* model [26]. Additionally, the Mg activation energy of Al_x_Ga_(1−x)_N alloy for 0 < x < 1 is set to scale linearly from 170 meV to 510 meV [6]. The Shockley-Read-Hall (SRH) recombination life-time, radiative recombination coefficient, Auger recombination coefficient, and light extraction efficiency are set as 15 ns, 2.13 × 10^−11^ cm^3^/s, 2.88 × 10^−30^ cm^6^/s, and 15%, respectively [27]. Moreover, the built-in polarization due to spontaneous and piezoelectric polarization is estimated using the methods proposed by Fiorentini et al. [28] and considered as 50% of the theoretical value. All simulations are performed at room temperature and other band parameters can be found elsewhere [29].

## 3. Results

**Figure 2:**
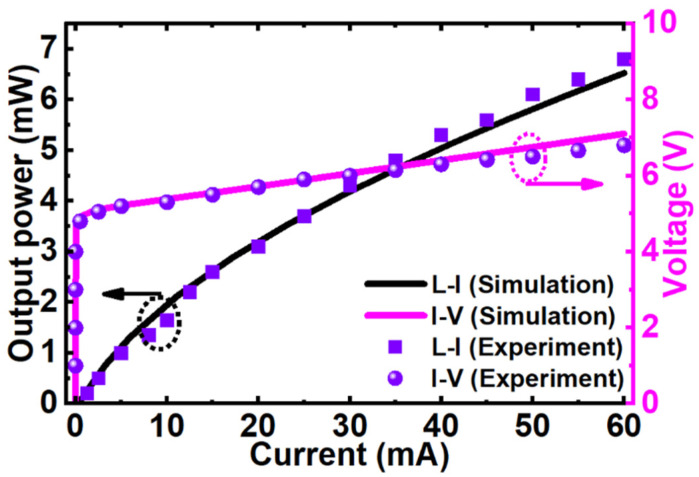
Measured and calculated light-current-voltage characteristics of LED 1 for model validation [21].

The numerical device model and parameters implemented in this study are optimized based on the experimentally measured data of LED 1 published by Yan et al. [21]. Figure 2 shows the numerically calculated light-current-voltage curves of LED 1 closely matching with the experimentally obtained curves that validate our device model.

**Figure 3:**
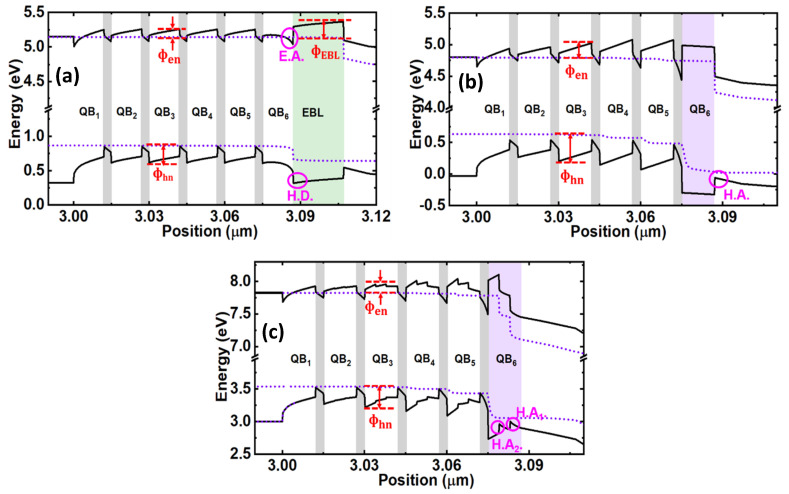
Energy band diagram of (**a**) LED 1, (**b**) LED 2, and (**c**) LED 3 at an injection current of 60 mA. E.A. is the electron accumulation region, H.D. is the hole depletion region, and H.A. is the hole accumulation region.

To investigate the performance of the proposed structure, we have performed a numerical study on three LEDs, and the results are carefully analyzed. As a part of this study, we have calculated the energy-band diagrams of LED 1, LED 2, and LED 3 at 60 mA injection current, as shown in Figure 3. The effective CBBH at the corresponding barrier (n) and EBL layer are denoted as ф_en_ and ф_EBL_, respectively.

**Figure 4:**
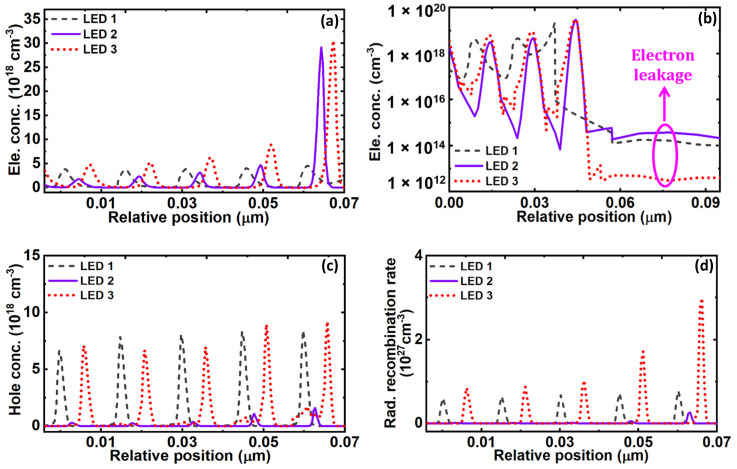
(**a**) Electron concentration, (**b**) electron leakage, (**c**) hole concentration, and (**d**) radiative recombination of LED 1, LED 2, and LED 3.

In the same way, ф_hn_ denotes the effective valence band barrier heights (VBBH) at the corresponding barrier (n). The values for each of CBBH are extracted from the energy band diagrams and listed in Table 1. The value of ф_EBL_ is 235 meV in the case of LED 1 due to EBL, which is the maximum CBBH to block the electron overflow in LED 1. This value is comparatively low in contrast to LED 2 and LED 3 without EBL. In LED 2 and LED 3, the value of ф_en_ is progressively increasing with each QB and effectively blocking the electrons overflow by preventing the electrons from jumping out of the QWs. Further, the value of maximum CBBH i.e., ф_e6_ in LED 3, is even higher than that of LED 2 as listed in Table 1, demonstrating LED 3 is the optimal choice to confine the electrons in the active region. As a result, in comparison with other LEDs, improved and maximum electron concentration in the active region for LED 3 was observed and is shown in Figure 4a. Though LED 2 has boosted electron concentration as compared to LED 1, but it is lower than LED 3. It is also noticed that due to improved electron confinement in the active region, electron leakage into the *p*-region is significantly reduced in LED 3, as shown in Figure 4b. Subsequently, this would reduce the non-radiative recombination of the overflowed electrons, and incoming holes in the *p*-region thereby contribute to better hole injection efficiency into the active region. However, LED 2 has even higher electron leakage as compared to LED 1. Due to this, the non-radiative recombination in the *p*-region of LED 2 would increase and reduce the hole injection efficiency into the active region, irrespective of the creation of negative sheet polarization charges at the last QB and *p*-Al_0.5_Ga_0.5_N interface.

It is worthwhile to note that in the last QB of LED 1, a sharp bending in the conduction band is formed due to induced positive polarization sheet charges at the heterointerface of the last QB and EBL. This area accumulates a large number of electrons i.e., ~ 3.66 × 10^16^ cm^−3^, which eventually contributes to non-radiative recombination [30]. In addition, due to this induced positive polarization sheet charges in LED 1, a hole depletion region is formed at the heterointerface of the last QB and EBL, as shown in Figure 3a, which reduces the hole injection efficiency [5]. The formation of the hole depletion region problem is eliminated in the case of LED 2 and LED 3 by removing the EBL. In the case of LED 2, a hole accumulation region is formed at the interface of the last QB and *p*-region, which generally should improve the hole injection efficiency, whereas in LED 3 the hole injection efficiency even should further improve due to the formation of two-hole accumulation regions as shown in Figure 3c. The boosted hole injection efficiency in LED 3 can be seen from Figure 4c. This is also because of the reduced electron overflow in LED 3 due to improved electron confinement in the active region. Moreover, the effective VBBH, ф_hn_ due to each QB, are calculated and listed in Table 2. As ф_hn_ increases with the increase in Al composition in the QBs, the values of ф_hn_ are found to be high in LED 2 and LED 3 compared to LED 1. This supports the improved hole confinement and increased hole concentration in the active region. However, a very high ф_hn_ can also affect the hole transportation in the active region at the same time, which is in the case of LED 2. Moreover, already the hole injection efficiency is poor in LED 2, altogether the hole concentration is very low in LED 2, as shown in Figure 4c. In this regard, LED 3 has a smaller value of ф_hn_ as compared to LED 2 due to again GSQB structures. Altogether, due to effective hole injection efficiency along with a comparable height of ф_hn_ in the active region, hole concentration in LED 3 is relatively evenly distributed as compared to other LEDs. Overall, the hole concentration in the active region of all three LEDs is 7.2 × 10^16^ cm^−3^, 4.8 × 10^15^ cm^−3^, 7.8 × 10^16^ cm^−3^, respectively. Importantly, the overlap level of electron and hole wave functions in the active region for LED 1 and LED 3 are summarized in Table 3. It is seen that even though the hole concentration of LED 3 is close to LED 1, the proposed structure in LED 3 improves the electron and hole wavefunctions overlap level as compared to LED 1. As a result, the radiative recombination is significantly increased in LED 3, as depicted in Figure 4d. 

**Figure 5:**
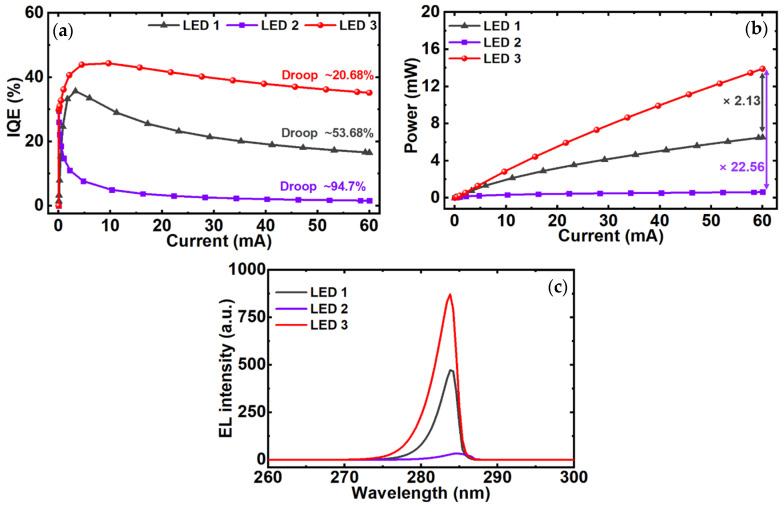
(**a**) Internal quantum efficiency (IQE), (**b**) output power, and (**c**) electroluminescence (EL) intensity of LED 1, LED 2, and LED 3.

Finally, the IQE and output power of LED 1, LED 2, and LED 3 as a function of injection current are illustrated in Figure 5a,b, respectively. Figure 5c depicts the electroluminescence (EL) spectra of the three LEDs. As shown in Figure 5a, LED 3 exhibits the maximum IQE of 44.34%, whereas it is only 35.69% and 29.46% in the case of LED 1 and LED 2, respectively. In addition, the droop in the IQE during 0 mA–60 mA injection current is remarkably reduced to 20.68% in the proposed structure as compared to 53.68% and 94.7% in LED 1 and LED 2, respectively. This is due to the enhanced carrier transportation and confinement in the active region, thereby reduced electron overflow into the *p*-region because of GSQBs in the proposed structure. As depicted in Figure 5b, the output power of LED 3 is 2.13 times higher than LED 1 and 22.56 times higher than LED 2. As shown in Figure 5c, LED 3 depicts higher EL intensity as compared to LED 1 and LED 2 at the emission wavelength of ~284 nm due to improved radiative recombination in the active region. EL intensity of LED 3 is ~2.12 times higher than LED 1 and ~22.24 times higher than LED 2. Different parameters related to IQE and output power of three LED structures are summarized in Table 4.

**Figure 6:**
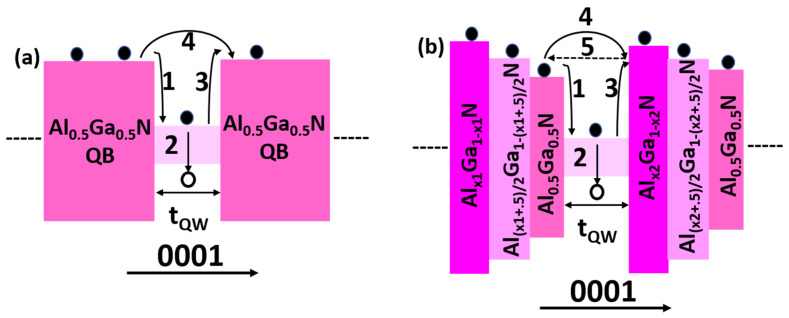
Schematic of Energy band diagram of (**a**) LED 1 and (**b**) LED 3.

To better understand the role of GSQB structure in LED 3, the schematic model for transportation of electrons in LED 1 and LED 3 is depicted in Figure 6. In this study, the total number of injected electrons into the *n*-Al_0.6_Ga_0.4_N region is considered as *N_0_* for LED 1 and LED 3. For the simplicity of the model, electron loss through non-radiative recombination in *n*-Al_0.6_Ga_0.4_N region is neglected. The captured electrons in the quantum well (*N_capture_*) are correlated with the electron mean free path (*l_MFP_*) as expressed in Equation (2) [31].

(2) $$N_{capture}=N_{0}\times [1-e^{\frac{-t_{QW}}{l_{MFP}}}]$$

where *t_QW_* is the quantum well thickness. Illustrated in Figure 6a,b, the incoming electrons (*N_0_*) are scattered and fall into the quantum wells, denoted by process 1. Some of those fallen electrons recombine with the holes radiatively as well as with the crystal defects as depicted by process 2, while remaining electrons escape from the QWs as illustrated by process 3. In addition, some electrons with longer *l_MFP_* travel to a remote position without being captured by the QWs as indicated by process 4. To increase *N_capture_,* the *l_MFP_* of these electrons needs to be reduced so that the electron concentration in the QWs would be increased that would favor the higher radiative recombination rate in the active region. At the same time, *l_MFP_* depends on thermal velocity (*v_th_*) and the scattering time (*τ_sc_*) as shown in Equation (3). For LED 1, *v_th_* can be further expressed as illustrated in Equation (4) [31].

(3) $$l_{MFP}=\upsilon_{th}\times \tau_{sc}$$

$$\upsilon_{th}=\sqrt{2\times \frac{\left[E+\Delta E_{c}+qV_{1}-\Delta E_{c}\right]}{m_{e}}}$$

(4) $$=\sqrt{2\times \frac{\left[E+qV_{1}\right]}{m_{e}}}$$

where *E* is the excess kinetic energy in the *n*−Al_0.6_Ga_0.4_N layer, *qV_1_* is the work done to the electrons by the induced polarization electric field in QBs of LED 1, and *m_e_* is the effective mass of electrons. +Δ*E_c_* denotes the conduction band offset between QB_n_ and QW_n_, while −Δ*E_c_* represents the conduction band offset between QW_n_ and QB_n + 1_. On the other hand, the GSQB structure in LED3 forms discontinuity in the conduction band of QB layers due to which the probability of the electrons to be scattered increases. 

**Figure 7:**
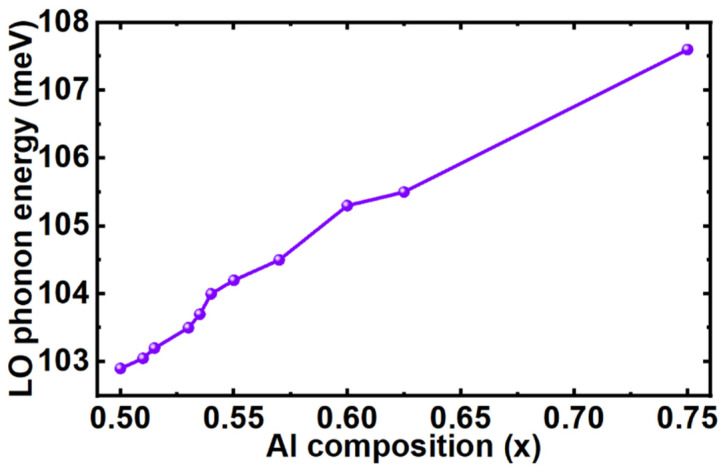
Calculated longitudinal optical (LO) phonon energy in Al_x_Ga_1−x_N layer.

Therefore, electrons would be thermalized more efficiently by interacting with longitudinal optical (LO) phonons, thereby reducing the *v_th_* and *l_MFP_,* as a result, electron confinement in the active region increases [19]. Hence, the *v_th_* in LED3 can be expressed as follows,

(5) $$\upsilon_{th}=\sqrt{2\times \frac{\left[E+\Delta E_{c1}+qV_{2}-\Delta E_{c2}-\hbar \omega_{LO}\right]}{m_{e}}}$$

(6) $$\hbar \omega_{LO}=\hbar \omega_{LO(step1)}+\hbar \omega_{LO(step2)}+\hbar \omega_{LO(step3)}$$

where +∆*E*_*c*1_ represents the conduction band offset between QB_n_ and QW_n_ whereas −∆*E*_*c*2_ is the conduction band offset between QW_n_ and QB_n + 1_. As the QB heights are varying in LED 3 along the growth direction, Δ*E*_*c*1_ − Δ*E*_*c*2_ can therefore not be eliminated. *qV_2_* is the work done to the electrons by the induced polarization electric field in GSQBs of LED 3. The −*ℏω_LO_* denotes the total energy loss by phonon emissions due to each step layer in GSQBs. The values of *qV* due to each QB in LED1 and LED3 are listed in Table 5. Further, the values of ℏ*ω_LO_* in each step of GSQBs for LED3 are calculated [32] and presented in Figure 7.

From Equations (4) and (5), it is understood that (*E* + Δ*E*_*c*1_ + *qV*_2_ − Δ*E*_c2_ − *ℏω_LO_*) < (*E + qV*_1_). Consequently, *v_th_* for LED3 would be less as compared to LED1. As a result, *l_MFP_* would be reduced, which improves the electrons capture (*N_capture_*) ability of the QWs in LED3. In addition, the electron overflow happening due to process 4 can also be reduced by increasing the barrier height as in the proposed structure shown in Figure 6b. Here, the QB heights before and after the QWs are not at the same level, rather it is progressively increasing along the growth direction due to which some of the electrons from process 3 and 4 would bounce back denoted as process 5, which can also aid to improved electron concentration in the QWs in comparison with LED 1 as shown in Figure 4a. The proposed AlGaN deep UV LEDs using graded staircase barriers can also be realized by experimentation due to a simple device architecture. As different AlGaN based UV LEDs with thinner epilayers than our proposed structure have already been grown by metal-organic chemical vapor deposition (MOCVD) [33,34,35] and molecular beam epitaxy (MBE) [36,37]. Therefore, it is anticipated that the proposed structure can also be grown by both MBE and MOCVD.

## 4. Conclusions

We have numerically demonstrated and investigated the performance of EBL-free AlGaN UV LEDs emitting light at ~284 nm wavelength with the incorporation of GSQB structures. The reduced thermal velocity and mean free path of electrons improved the electron capture efficiency in the multi QWs, thus electron overflow was suppressed eminently. In addition, carefully engineered GSQBs promoted the hole injection by forming negative sheet polarization charges and improved the spatial overlap of the electron-hole wavefunction. Therefore, the proposed structure exhibited higher radiative recombination and recorded output power of 13.9 mW at 60 mA injection current, which is 2.13 times higher than the conventional structure. Hence, the reported structure shows incredible potential to develop high-efficiency UV light emitters for real-world applications.

## Figures and Tables

**Table 1:** Effective conduction band barrier heights (CBBH) of QBs (ф_en_) and EBL (ф_EBL_) for LED 1, LED 2, and LED 3.

Conduction Band Barrier Heights (CBBH)	LED 1	LED 2	LED 3
ф_e2_	114.3 meV	167.3 meV	104.42 meV
ф_e3_	113.8 meV	232.6 meV	134.77 meV
ф_e4_	112.6 meV	300.6 meV	191.15 meV
ф_e5_	110.1 meV	330.1 meV	230.22 meV
ф_e6_	31.2 meV	242.2 meV	322.54 meV
ф_EBL_	235 meV	-	-

**Table 2:** Effective valence band barrier heights (VBBH) of QBs (ф_hn_) for LED 1, LED 2, and LED 3.

Valence Band Barrier Heights (VBBH)	LED 1	LED 2	LED 3
ф_h2_	251.9 meV	367.1 meV	269.16 meV
ф_h3_	250.3 meV	427.1 meV	321.19 meV
ф_h4_	249.3 meV	471.4 meV	368.15 meV
ф_h5_	248.1 meV	502.1 meV	406.93 meV

**Table 3:** Values of the electron and hole wave function spatial overlap levels in the active region for LED 1 and LED 3.

LEDs	1st QW (%)	2nd QW (%)	3rd QW (%)	4th QW (%)	5th QW (%)
LED 1	34.36	28.39	26.86	26.03	25.14
LED 3	33.77	34.96	34.94	32.77	29.32

**Table 4:** Comparison of internal quantum efficiency (IQE) and output power of LED 1, LED 2, and LED 3.

Parameters	LED 1	LED 2	LED 3
Max. IQE (%)	35.69 at 3.26 mA	29.46 at 0.04 mA	44.34 at 9.66 mA
IQE (%) at 60 mA	16.53	1.56	35.17
IQE (%) droop	53.68	94.7	20.68
Power at 60 mA (mW)	6.52	0.616	13.9

**Table 5:** Comparison of *qV1* and *qV2* values of LED 1 and LED 3.

LEDs	QB2	QB3	QB4	QB5
LED1	88.8 meV	87.2 meV	86.5 meV	85.0 meV
LED3	84.2 meV	100.6 meV	104.4 meV	78 meV
